# Biomarcadores na Avaliação de Pacientes Submetidos à Quimioterapia com Antraciclinas

**DOI:** 10.36660/abc.20230821

**Published:** 2024-02-22

**Authors:** Aurora Felice Castro Issa

**Affiliations:** 1 Instituto Nacional de Cardiologia Rio de Janeiro RJ Brasil Instituto Nacional de Cardiologia, Rio de Janeiro, RJ – Brasil

**Keywords:** Biomarcadores Farmacológicos/dosagem, Neoplasias, Tratamento Farmacológico/efeitos adversos, Antraciclinas/uso terapêutico

As antraciclinas são componentes importantes de muitos regimes de quimioterapia para várias malignidades sólidas e hematológicas, incluindo câncer de mama, ginecológico e de bexiga, bem como leucemia e linfoma. Infelizmente, estão associadas a um risco reconhecido de cardiotoxicidade.^1^ Os medicamentos desta classe incluem doxorrubicina, daunorrubicina, epirrubicina, mitoxantrona e idarrubicina. As evidências disponíveis sugerem que a lesão cardíaca induzida pela antraciclina ocorre durante a exposição e evolui ao longo do tempo.^2^

A disfunção cardíaca relacionada à terapia do câncer (DCRTC), a cardiotoxicidade clássica associada às antraciclinas, é um evento adverso grave que leva à interrupção do tratamento do câncer, à insuficiência cardíaca (IC) potencialmente grave e até à morte. A identificação precoce da disfunção cardíaca relacionada à terapia do câncer é essencial, pois permite a implementação precoce da terapia médica para IC.

Atualmente, não existe uma definição universal para toxicidade cardíaca induzida por antraciclina (TCIA). O diagnóstico é feito devido a IC de início recente ou evidência de imagem de disfunção ventricular esquerda (VE), que é comumente caracterizada por uma diminuição de ≥ 10% na fração de ejeção do VE para um valor menor que o limite inferior do normal.^2^ Uma redução relativa de > 15% na deformação longitudinal global do VE é um parâmetro útil para prever a TCIA, uma vez que o comprometimento da deformação miocárdica precede a disfunção contrátil.^3^ A abordagem diagnóstica disponível carece de sensibilidade para detectar disfunção cardíaca subclínica precoce e não pode prever com segurança resultados futuros. Um modelo preditivo melhorado seria capaz de detectar TCIA antes da diminuição da FEVE e do início dos sintomas.

Michelettie et al.,^4^ demonstraram a existência de uma rede pró-inflamatória desencadeada pela quimioterapia do câncer de mama que poderia aumentar a permeabilidade dos cardiomiócitos, permitir o extravasamento de proteínas circulantes e induzir a produção de um marcador inflamatório como a Proteína C Reativa altamente sensível, o que evidencia o papel potencial dos biomarcadores na identificação do TCIA.^4^

A literatura sobre o uso de biomarcadores para estratificação de risco de DCRTC antes da terapia oncológica é limitada e as recomendações baseiam-se principalmente na opinião de especialistas. Neste momento, considera-se que a troponina e os peptídeos natriuréticos apresentam benefícios potenciais se forem dosados durante o tratamento, apesar dos achados controversos.^5,6^ Mas, na verdade, os pontos de corte geralmente aceitos e os valores de referência dos biomarcadores CV não foram estabelecidos para pacientes com câncer ou para aqueles que recebem terapias contra o câncer. Além disso, os níveis de NP e cTn podem diferir de acordo com os laboratórios locais e podem ser alterados por muitos fatores, incluindo idade, sexo, função renal, obesidade, infecções e comorbidades.^7^ A literatura também é limitada, especialmente em outros novos biomarcadores.

Recentemente, Dean et al. examinaram os níveis de biomarcadores cardíacos e não cardíacos antes, após a última dose e 3–6 meses após o término da quimioterapia com doxorrubicina e identificaram biomarcadores com alterações significativas de intervalo em resposta à terapia com antraciclina.^8^

Nesta edição foram apresentados os resultados de um estudo caso-controle que comparou os valores de diferentes biomarcadores cardiovasculares que foram medidos em pacientes com câncer de mama após o último ciclo de tratamento com doxorrubicina e em pacientes sem câncer de mama ou doença cardiovascular. Foram encontradas algumas diferenças nos resultados, mas não são definitivas sobre o uso dos estudos de biomarcadores.^9^ Apesar disso, esses resultados preliminares podem ser importantes para estudos futuros.

A procura de testes que melhorem a avaliação e o tratamento dos pacientes em cardio-oncologia está ligada ao objetivo global desta disciplina, que é permitir que os pacientes com câncer recebam os melhores tratamentos oncológicos possíveis com segurança, minimizando a toxicidade cardiovascular relacionada com a terapia oncológica.^10^ Estudos de validação em diferentes cenários clínicos e de custos sobre os biomarcadores são essenciais.

## References

[1] Vejpongsa P, Yeh ET (2014). Prevention of Anthracycline-Induced Cardiotoxicity: Challenges and Opportunities. J Am Coll Cardiol.

[2] Cardinale D, Iacopo F, Cipolla CM (2020). Cardiotoxicity of Anthracyclines. Front Cardiovasc Med.

[3] Thavendiranathan P, Poulin F, Lim KD, Plana JC, Woo A, Marwick TH (2014). Use of myocardial strain imaging by echocardiography for the early detection of cardiotoxicity in patients during and after cancer chemotherapy: a systematic review. J Am Coll Cardiol.

[4] Micheletti P, Silva JC, Scandolara T, Kern R, Alves VD, Malanowski J (2021). Proinflammatory circulating markers: new players for evaluating asymptomatic acute cardiovascular toxicity in breast cancer treatment. J Chemother.

[5] Lyon AR, Fernández TL, Couch LS, Asteggiano R, Aznar MC, Klein JB (2022). 2022 ESC Guidelines on cardio-oncology developed in collaboration with the European Hematology Association (EHA), the European Society for Therapeutic Radiology and Oncology (ESTRO) and the International Cardio-OncologySociety(IC-OS). Eur Heart J.

[6] Sendón J, Ortega C, Auñon P, Soto A, Lyon AR, Farmakis D (2020). Classification, prevalence, and outcomes of anticancer therapy-induced cardiotoxicity: the CARDIOTOX registry. Eur Heart J.

[7] Pudil R, Mueller C, Čelutkienė J, Henriksen PA, Lenihan D, Dent S (2020). Role of serum biomarkers in cancer patients receiving cardiotoxic cancer therapies: a position statement from the Cardio-Oncology Study Group of the Heart Failure Association and the Cardio-Oncology Council of the European Society of Cardiology. Eur J Heart Fail.

[8] Dean M, Kim MJ, Dimauro S, Tannenbaum S, Graham G, Liang BT (2023). Cardiac and noncardiac biomarkers in patients undergoing anthracycline chemotherapy - a prospective analysis. Cardiooncology.

[9] Pestana RMC, Silvino JPP, Oliveira AN, Soares CE, Sabino AP, Simões R (2023). New Cardiovascular Biomarkers in Breast Cancer Patients Undergoing Doxorubicin-Based Chemotherapy. Arq Bras Cardiol.

[10] Lancellotti P, Suter TM, Fernández T, Galderisi M, Lyon AR, Van Der Meer P (2019). Cardio-oncology services: rationale, organization, and implementation. Eur Heart J.

