# Supplementary material for: Biomarcadores na Avaliação de Pacientes Submetidos à Quimioterapia com Antraciclinas
Source: Arq Bras Cardiol. 2024 Feb 22;120(12):e20230821. [Article in Portuguese] doi: 10.36660/abc.20230821 (PMC11098577; doi:10.36660/abc.20230821)
Supplement: Translation

# Translation: Biomarkers in the Evaluation of Patients Undergoing Chemotherapy with Anthracyclines

**Authors:** Aurora Felice Castro Issa

**Affiliations:** 1 Rio de Janeiro RJ Brazil Instituto Nacional de Cardiologia, Rio de Janeiro, RJ – Brazil

**Keywords:** Biomarkers Pharmacological/dosage, Neoplasms, Drug Therapy/adverse effects, Anthracyclines/therapeutic use

Anthracyclines are important components of many chemotherapy regimens for various solid and hematologic malignancies, including breast, gynecologic, and bladder cancers, as well as leukemia and lymphoma. Unfortunately, they are associated with a recognized risk of cardiotoxicity.^1^ Drugs in this class include doxorubicin, daunorubicin, epirubicin, mitoxantrone and idarubicin. The available evidence suggests that anthracycline-induced injury to the heart occurs during exposure and evolves over time.^2^

Cancer therapy-related cardiac dysfunction (CTRCD), the classic cardiotoxicity associated with anthracyclines, is a serious adverse event that leads to interruption of cancer treatment, potentially severe heart failure (HF), and even death. Early identification of cancer therapy-related cardiac dysfunction is essential as it allows for early implementation of medical HF therapy.

Currently, there is no universal definition for anthracycline-induced cardiac toxicity (AICT). Diagnosis is made due to new-onset HF or imaging evidence of left ventricular (LV) dysfunction, which is commonly characterized by a ≥ 10% decrease in LV ejection fraction to a value less than the lower limit of normal.^2^ A relative reduction of > 15% in LV global longitudinal strain is a useful parameter to predict AICT as impairment in myocardial deformation precedes contractile dysfunction.^3^ The available diagnostic approach lacks the sensitivity to detect early subclinical cardiac dysfunction and cannot reliably predict future outcomes. An improved predictive model would be able to detect AICT prior to decreases in LVEF and symptom onset.

Micheletti et al.^4^ demonstrated the existence of a proinflammatory net triggered by breast cancer chemotherapy that could increase cardiomyocyte permeability, allow the leakage of circulating proteins, and induce the production of an inflammatory marker as highly sensitive C Reactive Protein, which evidences the potential role of biomarkers in the identification of AICT.^4^

The literature on the use of biomarkers for CTRCD risk stratification before cancer therapy is limited, and recommendations are mostly based on expert opinion. At this moment, troponin and natriuretic peptides are considered to have potential benefits if they are going to be measured during the treatment, despite controversial findings.^5,6^ But, in fact, generally accepted cut-offs and reference values of CV biomarkers have not been established for patients with cancer or for those who receive cancer therapies. In addition, levels of NP and cTn may differ according to local laboratories and may be altered by many factors, including age, sex, renal function, obesity, infections, and comorbidities.^7^ The literature is also limited specially in other novel biomarkers.

Recently, Dean et al. examined the levels of cardiac and noncardiac biomarkers before, after the last dose of, and 3–6 months after completion of doxorubicin chemotherapy and identified biomarkers with significant interval changes in response to anthracycline therapy.^8^

In this issue was presented the results of a case-control study that compared the results of different cardiovascular biomarkers that were measured in patients with breast cancer after the last cycle of treatment with doxorubicin and in patients without breast cancer or cardiovascular disease. Some differences in the results were found, but they are not definitive about the use of the biomarkers studies.^9^ Despite that, these preliminary results can be important for future studies.

The search for tests that improve the evaluation and treatment of patients in cardio-oncology is linked to the overarching goal of this discipline, which is to allow patients with cancer to receive the best possible cancer treatments safely, minimizing cancer therapy-related cardiovascular toxicity during cancer care.^10^ Validation studies in different clinical scenarios and studies about the costs of the biomarkers will be essential.
